# Clinical comparison of unilateral biportal endoscopic transforaminal lumbar interbody fusion verse 3D microscope-assisted transforaminal lumbar interbody fusion in the treatment of single-segment lumbar spondylolisthesis with lumbar spinal stenosis: a retrospective study with 24-month follow-up

**DOI:** 10.1186/s13018-023-04401-4

**Published:** 2023-12-08

**Authors:** Wenlong Guo, Tong Li, Chaoqun Feng, Yang Yu, Youpeng Hu, Xiaohong Fan

**Affiliations:** 1 Chengdu University of Traditional Chinese Medicine, Chengdu, 610000 China; 2 Department of Orthopaedics, Hospital of Chengdu University of Traditional Chinese Medicine, Chengdu, 610075 China

**Keywords:** Degenerative lumbar spondylolisthesis, Minimally invasive surgery, Lumbar interbody fusion, Unilateral biportal endoscopy, Spinal surgery

## Abstract

**Objective:**

To explore the safety and the mid-term efficacy of unilateral biportal endoscopic transforaminal lumbar interbody fusion (UBE-TLIF) and 3D microscope-assisted transforaminal lumbar interbody fusion (MMIS-TLIF) for treating single-segment lumbar spondylolisthesis with lumbar spinal stenosis (DLS-LSS).

**Methods:**

The clinical data of 49 patients who underwent UBE-TLIF or MMIS-TLIF in our hospital were retrospectively analyzed, including 26 patients who underwent the UBE-TLIF and 23 patients who underwent the MMIS-TLIF. The demographic and perioperative outcomes of patients before and after surgery were reviewed. Visual analogue scale (VAS) and Oswestry disability index (ODI) were used to evaluate the clinical outcomes of patients before surgery and at 1, 3, 6, 12 and 24 months after surgery. The lumbar lordosis angle (LL), disc height (DH) and lumbar intervertebral fusion rate were assessed before surgery and at the last follow-up.

**Results:**

The VAS and ODI scores of the two groups were improved compared with those before surgery. The ODI of UBE-TLIF group was lower than that of MMIS-TLIF group at 1, 3, 6, and 12 months after surgery, and there were no significant differences between the two groups at other time points (*P* > 0.05). There were no significant differences in VAS between the two groups at each time point (*P* > 0.05). However, the UBE-TLIF group had more advantages in blood loss and hospital stay. The complications between the UBE-TLIF group (11.54%) and the MMIS-TLIF group (17.39%) were comparable (*P* > 0.05). Radiographic outcomes showed that the LL and DH of the two groups were improved compared with those before surgery, and the difference before and after surgery was not significant (*P* > 0.05). The fusion rate was 96.2% in the UBE-TLIF group and 95.7% in the MMIS-TLIF group. There was no significant difference in the fusion rate between the two groups (*P* > 0.05).

**Conclusions:**

Both UBE-TLIF and MMIS-TLIF have favorable outcomes for treating single-segment DLS-LSS. Both groups have the advantages of clear surgical vision, high surgical efficiency, and favorable mid-term efficacy. In addition, compared with MMIS-TLIF, UBE-TLIF causes less intraoperative bleeding and faster postoperative recovery.

## Background

Degenerative lumbar spondylolisthesis (DLS) refers to the relative displacement of two vertebral bodies, that leads to spinal instability, and compression of the corresponding nerves to produce chronic and persistent low back or radicular leg pain. Clinically, it is often combined with lumbar spinal stenosis (LSS) [1, 2]. It is generally believed that surgical treatment is often considered if conservative treatment fails [3, 4]. Lumbar interbody fusion (TLIF) is considered a common surgical method for the treatment of DLS-LSS [5, 6]. However, traditional open TLIF causes greater damage to paravertebral soft tissues and more bleeding [7, 8]. With the development of minimally invasive spine technology and the deepening of the concept, minimally invasive surgery transforaminal interbody fusion (MIS-TLIF) has gained increased attention in the spinal community because of its minimal trauma, less intraoperative blood loss and favorable clinical efficacy [9]. However, the narrow working channel is a problem in MIS-TLIF surgery and often leads to a limited visual field [10]. Therefore, some researchers have proposed TLIF combined with endoscopy or 3D microscopy. The advantage is that the microscope can provide a clear surgical vision, thereby improving surgical efficiency [11].

Unilateral biportal endoscopy (UBE) is being favored by an increasing number of spinal surgeons owing to its unique advantages. Compared with coaxial single-portal spinal endoscopy, UBE has a working portal and a viewing portal, and thus has broader surgical vision and operational flexibility, which undoubtedly improves the efficiency of surgery [12, 13].

Studies have demonstrated that UBE-TLIF for treating DLS-LSS also has the advantages of less trauma and bleeding [14]. Although UBE technology has received increasing attention, few studies have compared UBE with other minimally invasive procedures for DLS-LSS, and relatively long-term follow-up results are lacking. This study retrospectively analyzed the clinical data of patients who underwent UBE-TLIF for single-segment DLS-LSS in our hospital, and compared them with those of patients who underwent MMIS-TLIF during the same period to explore the clinical efficacy of UBE-TLIF in the treatment of single-segment DLS-LSS and report the surgical techniques and perioperative complications of UBE-TLIF in the treatment of single-segment DLS-LSS.

## Methods

### Patient data

We retrospectively reviewed the clinical data of 49 patients diagnosed with DLS-LSS, who underwent UBE-TLIF or MMIS-TLIF at our hospital between September 2019 and March 2021. The inclusion criteria were as follows: (1) patients with low back pain or radicular leg pain, with or without intermittent neurological claudication, and a radiograph or computed tomography (CT) scan showing meyerding grade I or II lumbar spondylolisthesis with LSS; (2) absence of improvement after conservative treatment for at least 3 months; and (3) follow-up time of more than 2 years and complete data. The exclusion criteria were as follows: (1) lumbar tuberculosis, tumor, infection, or trauma; (2) osteoporosis,* T* value less than − 2.5 [15]; and (3) prior lumbar surgery. According to the inclusion and exclusion criteria, 49 patients who met the criteria were included in this study, of whom 26 were treated with UBE-TLIF and 23 with MMIS-TLIF.

### Surgical procedures

**Fig. 1:**
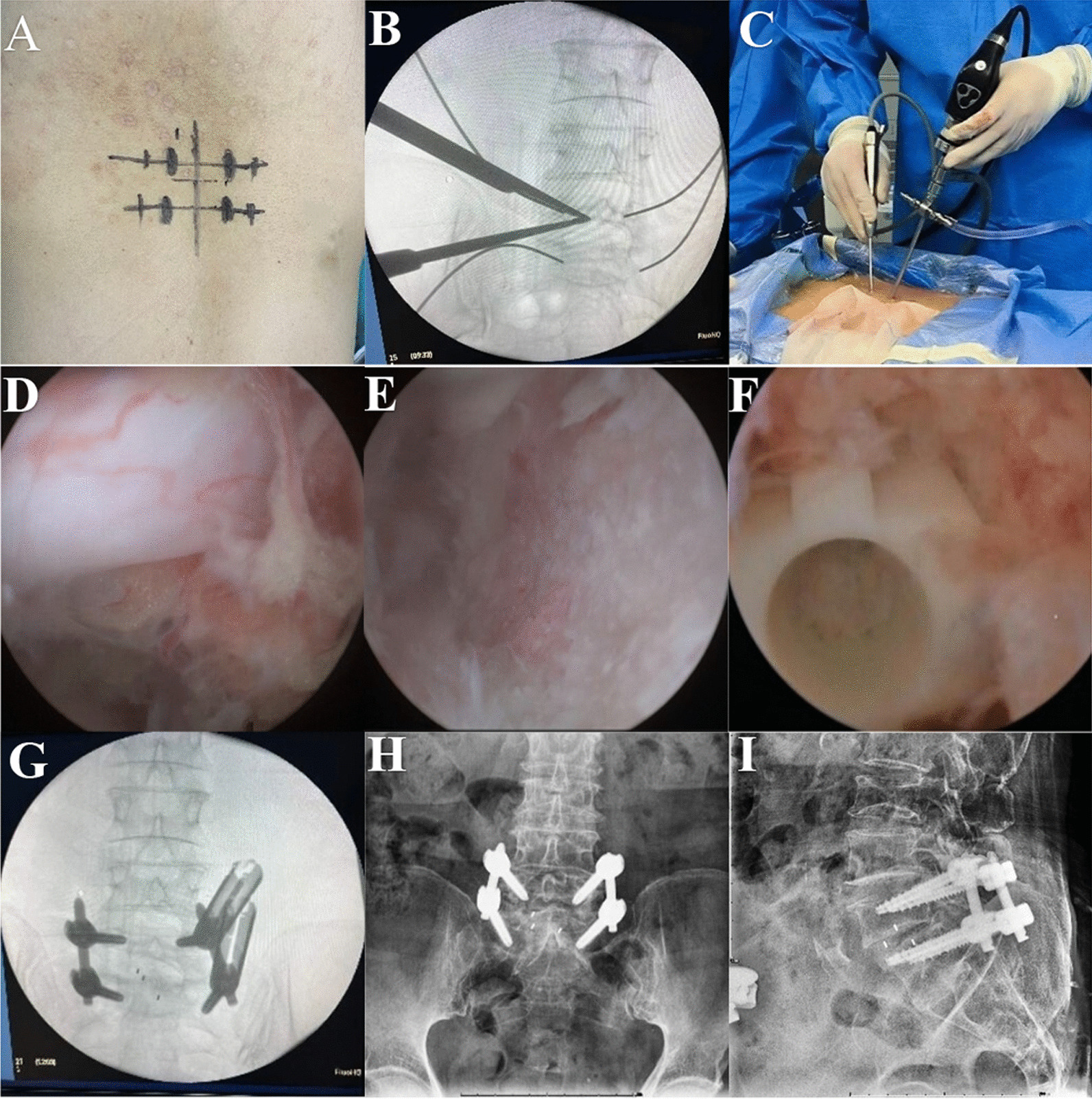
Unilateral biportal endoscopy lumbar interbody fusion (UBE-TLIF) surgical procedures: **A** Mark the lesion segment. **B** C-arm fluoroscopy to reconfirm surgical site. **C** Surgical site.** D** Endoscopic view of the relaxed neural structure after decompression. **E** Endplate preparation is completed. **F** A cage is placed under endoscopic guidance. **G** The position of the pedicle screw was confirmed again under fluoroscopy. **H** and **I** Images of final internal fixation

UBE-TLIF: After general anesthesia, the patient was placed in the prone position. After routine disinfection and sterile sheeting, the C-arm fluoroscopy was used to identify the target vertebra, the insertion point of the affected vertebral pedicle was marked, and four guide needles were inserted percutaneously along the pedicle. Using the right approach as an example, two oblique incisions were made approximately 1.5 cm from the midline of the spine at the lower edge of the upper endplate and the upper edge of the lower endplate. The lower incision served as the endoscopic visual field channel, whereas the upper incision served as the operating channel for the surgical instrument. A serial dilator was used to gradually expand the incision and the subcutaneous tissue. An osteotomy and grinding drill were used to remove the inferior articular process from the outside to the inside; and then, the superior articular process was removed. The excised lamina and articular process were used as autologous bone. Part of the ligamentum flavum was removed to expose the intervertebral discs. Any tissue compressing the spinal cord or nerve roots was removed. The overlying cartilage was then removed, and the hard subchondral bone was preserved to prepare the upper and lower endplates. Endoscopic insertion of the intervertebral space confirmed that the endplate cartilage had been removed, and an appropriately sized cage was selected. Decompression of the spinal canal was checked to remove the occult compression. The allogeneic or autologous bone was compressed around the cage. Subsequently, the percutaneous pedicle screws were fixed under C-arm guidance (Fig. 1).

MMIS-TLIF: After general anesthesia, the patient was placed in the prone position, followed by routine disinfection and sterile sheeting. The incision was made in the paracentral part of the spinous process of the lesion segment and approximately 3 cm long on both the right and left sides. The skin and subcutaneous tissues were incised, and the small joints and transverse process roots on both sides of the lesion segment were exposed using a muscle space approach. The expansion channel was placed at the small joint and fixed to the free arm. The 3D microscope was connected to remove the lower articular process, and part of the upper articular process with the help of the microscope and the next process is similar to UBE-TLIF.

### Post‑operative management

The two groups of patients received postoperative prophylactic antibiotics, an intravenous infusion of non-steroidal anti-inflammatory drugs, and anti-inflammatory analgesics. On the second postoperative day, the drainage tube was removed at noon, and patients were encouraged to stay in bed for functional exercises in the afternoon. On the third day after surgery, if the patient's pain was effectively relieved and no infection symptoms occurred, the patient was encouraged to get out of bed, stand, and wear a waist brace for 3 months after surgery.

The operation time, intraoperative blood loss, length of hospital stay, and hemoglobin (Hb) levels before surgery and 1 day after surgery were recorded and compared between the two groups. Postoperative complications included wound infection, dural tears, intracranial hypertension, epidural hematoma, and cage subsidence at the last follow-up (lumbar lateral radiograph showing that the fusion cage exceeded the upper or lower endplates as cage subsidence). VAS and ODI scores were obtained from patients preoperatively and at 1, 3, and 6 months, and 1 and 2 years after surgery to assess the improvement in patients' function. Radiographic outcomes were assessed using LL and DH, as shown on radiography and CT before surgery and at the final follow-up. The Suk classification was used to assess intervertebral fusion at the last follow-up, and the fusion rate was calculated as = (fusion cases + possible fusion cases) / total cases [16].

### Statistical analyses

SPSS27.0 (IBM Corporation, USA) was used to perform the statistical analyses. If the quantitative data met the criteria of normal distribution and homogeneity of variance, the *t*-test was used for analysis. If not consistent with normality and homogeneity of variance, the Mann–Whitney *U*-test was used for analysis. Continuous data were analyzed using repeated measures analysis of variance (ANOVA). Enumeration data were analyzed using the chi-squared test, and Ridit analysis was used for ranked data. Differences were considered statistically significant at *P* < 0.05.

## Results

The average age was 64.15 ± 6.42 years (range 54–78) in the UBE-TLIF group, and 66.09 ± 6.10 years (range 57–78) in the MMIS-TLIF group. There were no significant differences in baseline demographic indicators such as sex, age, disease course, surgical segment, type of spondylolisthesis, or Meyerding grade between the two groups (*P* > 0.05, Table 1). The operation parameters, including operation time, hospital stay, intraoperative blood loss, discrepancy between preoperative and postoperative Hb levels, and number of complications, are displayed in Table 2. UBE-TLIF was superior to MMIS-TLIF regarding intraoperative blood loss and length of hospital stay (*P* < 0.05).

**Table 1:** Baseline characteristics of the included patients

Characteristic		UBE-TLIF (*n* = 26)	MMIS-TLIF (*n* = 23)	*p* value
Sex				0.851
Male	12		10	
Female	14		13	
Age (years)	64.15 ± 6.42		66.09 ± 6.10	0.676
Course of disease (months)	15.43 ± 17.74		14.78 ± 14.79	0.758
Surgical segment				0.985
L3–4	5		4	
L4–5	12		11	
L5–S1	9		8	
Spondylolisthesis type				0.674
Isthmic spondylolisthesis	7		5	
Degenerative spondylolisthesis	19		18	
Meyerding grade				0.980
I	18		16	
II	8		7

**Table 2:** Perioperative data and complications

Characteristic	UBE-TLIF (*n* = 26)	MMIS-TLIF (*n* = 23)	*P* value
Operation time (mins)	144.19 ± 10.23	138.04 ± 13.31	0.335
Hospital stay (days)	8.31 ± 1.38	10.87 ± 2.72	0.002
Operation blood loss (ml)	170.15 ± 10.81	203.17 ± 14.57	0.017
Preoperative Hb (ml)	128.34 ± 10.37	127.39 ± 10.68	0.949
Postoperative 1d Hb (ml)	119.46 ± 10.57	116.87 ± 10.24	0.996
Hb discrepancy	8.88 ± 2.44	10.52 ± 3.99	0.002
Complications (yes/no)	3/23	4/19	0.861

**Fig. 2:**
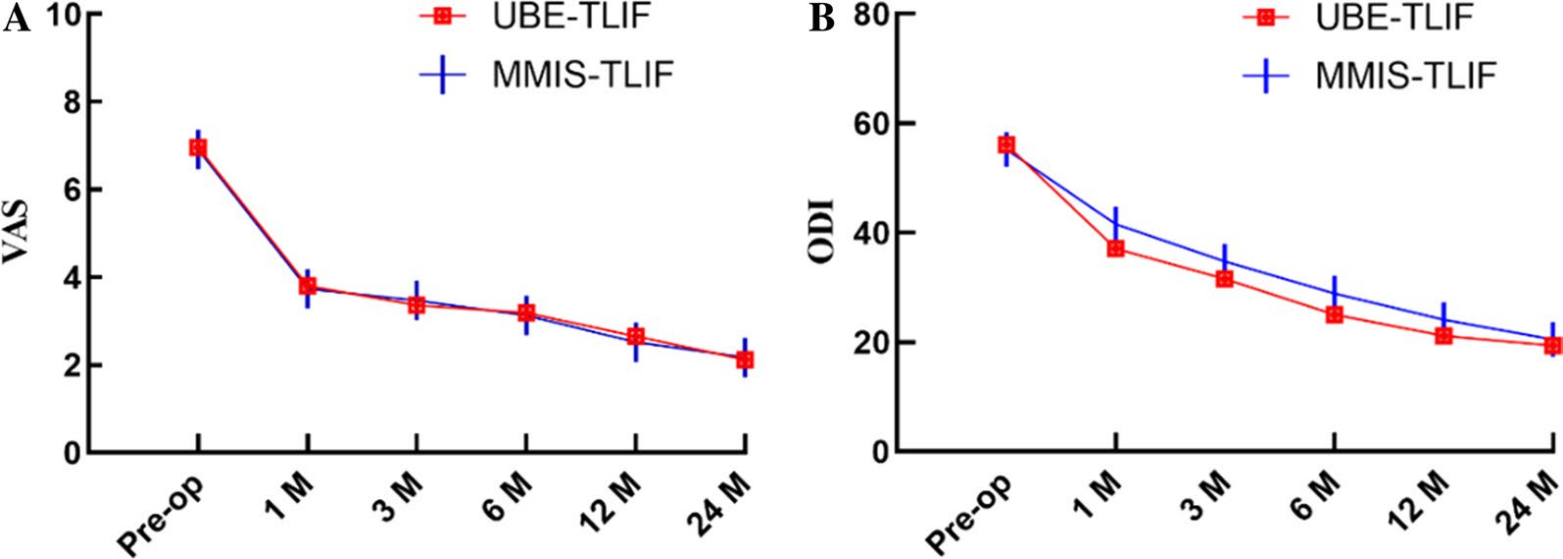
Clinical outcomes at specific follow-up time points. **A** Visual Analog Scale (VAS) score for both groups. **B** Oswestry Disability Index (ODI) score for both groups

There were no significant differences in the preoperative VAS and ODI scores between the two groups (*P* > 0.05). The VAS and ODI scores of the two groups at each time point after the operation were improved compared with those before the operation, and further improved with time; the difference was significant (*P* < 0.05). We observed no significant differences in the VAS scores between the two groups at any time point after surgery. At 1, 3, and 6 months and 1 year after surgery, the ODI score of the UBE-TLIF group was significantly lower than that of the MMIS-TLIF group(*P* < 0.05), and there was no statistically significant difference between the two groups at other time points (Fig. 2).

There were 3 complications in the UBE-TLIF group: 2 cases of dural tears and 1 case of intracranial hypertension. There were 4 complications in the MMIS-TLIF group, including 2 cases of wound infection, 1 case of dural tears and 1 case of intervertebral fusion cage subsidence. There was no significant difference in the complication rate between the two groups (11.54% vs. 17.39%) (*P* > 0.05).

**Fig. 3:**
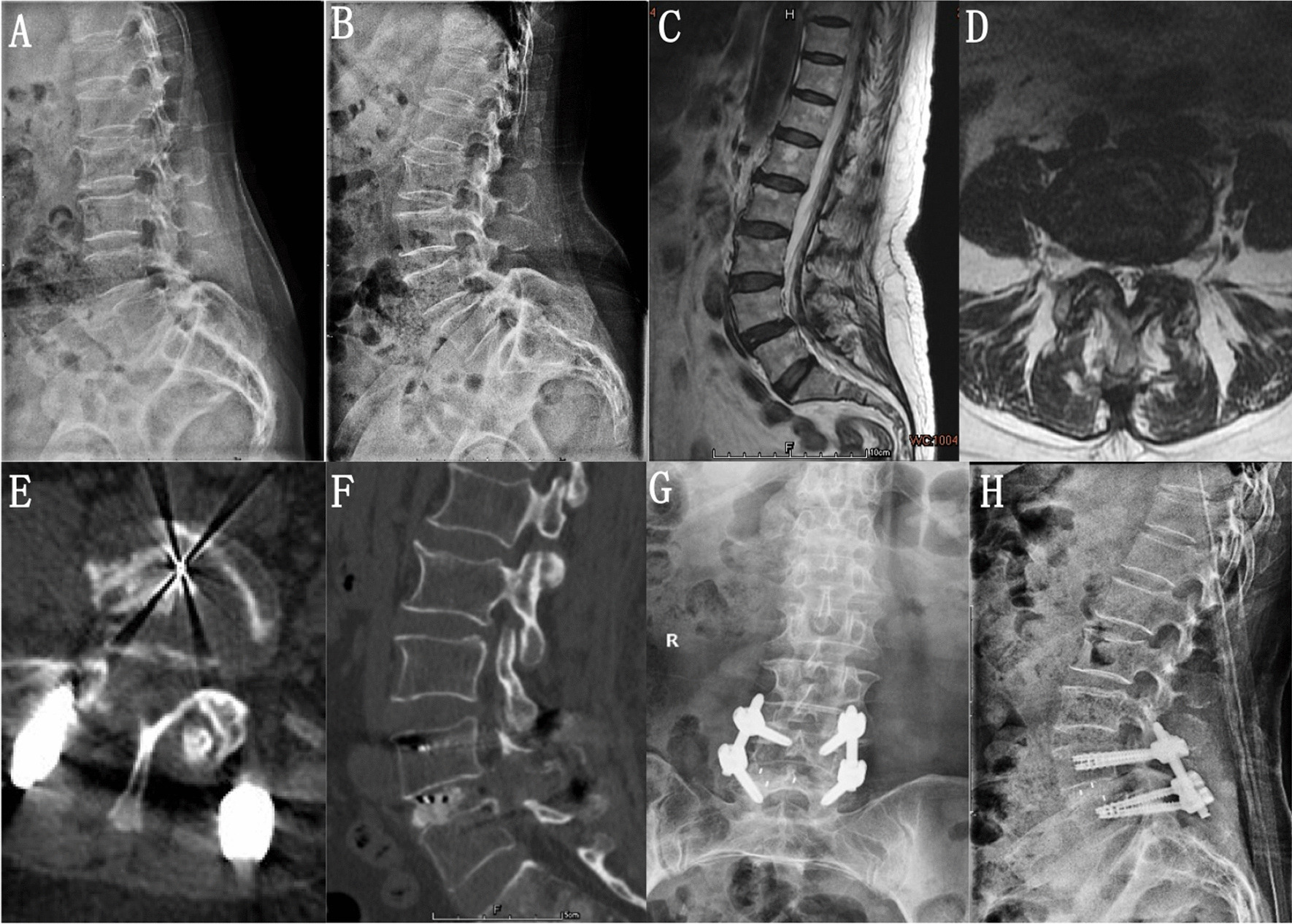
Case of a 65-year-old female patient who underwent unilateral biportal endoscopy lumbar interbody fusion (UBE-TLIF). **A**, **B** Preoperative dynamic radiography showed spondylolisthesis at the L4–5 level. **C**, **D** Preoperative magnetic resonance imaging (MRI) showed lumbar spinal stenosis at the L4–5 level. **E** Postoperative computed tomography (CT) showed unilateral laminectomy. **F** Postoperative CT showed that the cage position was good. **G**, **H** The last follow-up showed bone fusion between the vertebral bodies

At the last follow-up, the radiographic outcomes showed 23 UBE-TLIF intervertebral fusion cases, 2 possible fusion cases, 1 fusion failure case; and 20 MMIS-TLIF intervertebral fusion cases, 2 possible fusion cases, and 1 fusion failure case. There was no significant difference in the fusion rates between the two groups (96.2% vs. 95.7%) (*P* > 0.05). At the last follow-up, the LL and DH in the two groups improved compared to those before surgery, and there was no significant difference between the two groups (Tables 3, 4 and Fig. 3).

**Table 3:** Comparison of LL before and after operation

Characteristic	UBE-TLIF (*n* = 26)	MMIS-TLIF (*n* = 23)	*P* value
Preoperation	45.52 ± 1.69	46.19 ± 1.39	0.305
Last follow-up	48.27 ± 1.59	48.50 ± 1.19	–
Differentials	2.75 ± 1.05	2.30 ± 1.07	0.668
*P* value	0.000	0.000

**Table 4:** Comparison of DH before and after operation

Characteristic	UBE-TLIF (*n* = 26)	MMIS-TLIF (*n* = 23)	*P* value
Preoperation	8.64 ± 0.59	8.82 ± 0.55	0.844
Last follow-up	9.93 ± 0.94	10.13 ± 0.81	–
Differentials	1.29 ± 0.59	1.31 ± 0.63	0.891
*P* value	0.000	0.000

## Discussion

DLS is commonly encountered in clinical practice, particularly in older female patients. DLS can be divided into isthmic spondylolisthesis and degenerative spondylolisthesis according to the integrity of the pedicle isthmus. Based on the anatomical and clinical manifestations, patients with DLS often have LSS [17]. At present, it is generally believed that the treatment of LSS is to relieve nerve compression and restore the sequence and stability of the spine. In patients with DLS at the same time, interbody fusion and fixation based on decompression is often necessary. TLIF is considered the gold standard of interbody fusion surgery [18]. However, traditional open TLIF causes significant damage to the posterior spine, which easily affects spinal stability and is often accompanied by postoperative complications [19]. MIS-TLIF is a minimally invasive fusion surgery based on traditional TLIF and a minimally invasive channel. Studies have shown that MIS-TLIF has the same long-term efficacy as traditional surgery and has the advantages of less trauma, less intraoperative blood loss, and faster postoperative recovery [20, 21]. However, in clinical practice, surgical vision during MIS-TLIF is relatively narrow and unclear, which greatly affects the efficiency of the surgery. Therefore, our team tried to use a 3D microscope to assist MIS-TLIF, which not only solved the problem of narrow surgical vision during the operation but also improved the efficiency of the operation, reduced surgical trauma and intraoperative bleeding, and shortened the recovery time of postoperatively [22, 23].

Daniel first reported the new percutaneous endoscopic technique, UBE, in 1996. Unlike other endoscopes, UBE has two portals: an instrumentation portal and a viewing portal. With the help of a water medium and an arthroscope, clearer surgical vision and greater operative space can be achieved using the UBE technique [24]. Heo et al. [25] combined UBE technology with TLIF for the first time and found that UBE-TLIF significantly improved the VAS and ODI scores of patients. Kim et al. [26] used the UBE-TLIF technique to treat patients with lumbar spondylolisthesis and found that UBE-TLIF can also achieve a satisfactory fusion rate. We believe that the UBE technique can provide very clear surgical vision, and arthroscopy can be performed between the two endplates to observe the preparation process of the intervertebral space and endplate, providing the most suitable conditions for intervertebral fusion.

This study showed that there was no significant difference in the operation time between the two groups. The learning curve for UBE-TLIF is steep. Studies have shown that surgical techniques began to stabilize after 34 cases [27]. In the early stages of the learning curve, more time is required for surgery because of a lack of experience. When a surgeon has sufficient experience, the operative time is significantly reduced. UBE-TLIF is superior to MMIS-TLIF in hospital stay, intraoperative blood loss, and Hb discrepancy before and after the operation. This indicates that the UBE-TLIF technique can reduce damage to the soft tissues behind the spine. Continuous saline irrigation combined with radiofrequency electrocoagulation can minimize bleeding and provide clearer surgical vision [28]. Although 3D microscope-assisted MIS-TLIF can also provide clear surgical vision, the dissection of soft tissues behind the spine is still large, which undoubtedly increases the recovery time of patients and leads to longer hospital stays. In summary, both groups had the advantages of clear vision and high surgical efficiency; however, UBE-TLIF resulted in intraoperative bleeding and faster postoperative recovery.

The VAS and ODI scores of the two groups improved compared with those before surgery, and gradually improved over time. This shows that both UBE-TLIF and MMIS-TLIF can relieve clinical symptoms in patients. We found that the ODI of the UBE-TLIF group was lower than that of the MMIS-TLIF group at 1, 3, 6, and 12 months postoperatively; however, there was no difference between the two groups at other time points. We believe that this is because MMIS-TLIF causes greater damage to the posterior ligament complex of the spine and requires the use of minimally invasive channels to continuously stretch the paravertebral muscles during the operation, resulting in ischemic necrosis of some muscles and, ultimately, back syndrome failure. This also shows that UBE-TLIF can restore the quality of life and work of patients faster.

No serious complications occurred in either groups, and there was no significant difference in complications rates between the two groups. Two patients had dural tears in the UBE-TLIF group, and one patient in the MMIS-TLIF group had dural tears. We speculated that this was caused by accidental injury during the operation of the surgical instruments. We successfully used a gelatin sponge to compress the rupture, and no cerebrospinal fluid leakage occurred postoperatively. One patient had raised intracranial pressure in the UBE-TLIF group [29] and had a headache, vomiting, increased blood pressure, and increased heart rate. We believe that this was because the patient had a history of hypertension. There was more bleeding when the facet joint was removed during surgery. To ensure clear vision during the operation, the irrigation pressure was increased. The liquid then entered the spinal canal through a ruptured dural sac, resulting in an increase in intracranial pressure. To avoid this situation, the following should be considered: The water irrigation pressure should not be too high; we suggest that it should be controlled under 25–30 mmHg (approximately 3.99 kPa). After the operation, the patient’s head should be elevated, and the operation time should be as short as possible. One patient developed a wound infection in the MMIS-TLIF group. Based on our drug sensitivity test, cefoperazone sodium and sulbactam sodium were selected as the anti-infective treatments. After two days of treatment, a routine blood examination showed a decrease in white blood cell count and C-reactive protein levels. When the index was close to normal, the patient was instructed to continue oral antibiotics. The patient recovered and was discharged. At the last follow-up, the radiographic outcomes showed one case of cage subsidence in the MMIS-TLIF group. Theoretically, perfect endplate preparation is critical for ensuring fusion. Therefore we considered that this was caused by damage to the endplate during the operation, and the damage to the endplate would undoubtedly cause fusion failure. To avoid this, we should not be too violent when dealing with severely degenerated endplates to prevent endplate damage. Second, when placing the cage, we should enter along the intervertebral space inclination angle to prevent the cage from destroying the endplate.

Radiographic outcomes showed that the fusion rates of UBE-TLIF and MMIS-TLIF were 96.2% and 95.7%, respectively, which is consistent with previous studies [26, 28]. There was no significant difference in the fusion rate between the two groups, indicating that both surgical methods achieved a high fusion rate. At the last follow-up, the LL and DH of the two groups were significantly improved compared to before surgery, and there was no significant difference between the two groups. It also shows that both UBE-TLIF and MMIS-TLIF can improve spinal stability after surgery and help restore the normal sequence of the lumbar spine.

## Conclusion

Both UBE-TLIF and MMIS-TLIF are safe and effective for treating single-segment DLS-LSS. Both techniques provide clear surgical vision and high surgical efficiency. Compared to MMIS-TLIF, UBE-TLIF has the advantages of less intraoperative blood loss and faster postoperative recovery. However, this was a retrospective study, and the sample was limited. In the future, multi-center, large-sample prospective randomized controlled trials are needed for further verification.
